# A rare case of acute myocardial infarction with heart failure following hump-nosed viper bite in a Sri Lankan female

**DOI:** 10.1186/s41182-024-00645-w

**Published:** 2025-01-06

**Authors:** W. M. D. A. S. Wanninayake, Tilan Aponso, Manohari Seneviratne, Dhanapala Dissanayake

**Affiliations:** Medical Unit, National Hospital of Sri Lanka, Colombo, Sri Lanka

**Keywords:** Type 2 myocardial infarction, Heart failure, Hump nosed viper bite

## Abstract

**Background:**

Hump-nosed viper (Hypnale species) bites are an important cause of mortality and morbidity in southern India and Sri Lanka, accounting for 27 and 77% of venomous snake bites, respectively. Previously, we knew them to be moderately venomous snakes, primarily causing local envenomation. However, recent reports have indicated severe systemic envenomation incidents, which include hemostatic dysfunction, microangiopathic hemolysis, kidney injury, myocardial toxicity, and even death. The literature rarely reports cardiac manifestations from hump-nosed viper bites, and all reported cases show cardiac manifestations within hours of the snake bite. The literature did not report late presentations of cardiac manifestations. Here, we report a case of hump-nosed viper bite complicated with type 2 myocardial infarction and acute pulmonary oedema secondary to acute heart failure in a Sri Lankan female presented to the National Hospital of Colombo, Sri Lanka, on day 3 after the snake bite.

**Case presentation:**

A local hospital transferred a previously healthy 39-year-old female from Kegalle, Sri Lanka, to our hospital for further condition management. We identified the offending snake as a hump-nosed viper after she reported a history of snake bites 3 days ago. She complained of chest tightness on day 3 of the illness and was found to have acute heart failure precipitated by troponin-positive non-ST elevation myocardial infarction in initial investigations. We performed a CT coronary angiography along with a metabolic screening, revealing normal coronary arteries and a negative metabolic screening. Supportive therapy with loop diuretics and oxygen managed her condition, and a follow-up 2D echocardiogram revealed complete recovery of her cardiac function. She was asymptomatic 3 months into the follow-up. Therefore, we concluded that the case was a venom-induced type 2 myocardial infarction leading to heart failure with acute pulmonary oedema, as the CT coronary angiogram showed normal coronary arteries.

## Introduction

Hump-nosed viper, a pit viper categorized under the genus Hypnale, is widely distributed in Sri Lanka and southern India and is a highly venomous snake*.* There are three main species, named *Hypnale hypnale, Hypnale zara, and Hypnale nepa,* which are found in Sri Lanka [1]. For centuries, it was considered a non-venomous snake until 1821, when hump-nosed viper bites in animals were reported to cause bleeding and swelling [2]. Literature on hump-nosed viper bites was scarce until the twentieth century. The unpredictability of developing severe envenomation and complications, according to the cases reported, suggests the importance of further studies to understand the lethal nature associated with hump-nosed viper bites. Currently, no antivenom is available for hump-nosed viper bites in Sri Lanka or India. In addition to local envenomation, out of the systemic manifestations, venom-induced consumptive coagulopathy and renal failure are the most reported systemic effects. Rarely, cardiac manifestations like myocardial infarction [3] and atrial fibrillation [4] are being reported in the literature.

We report a case of venom-induced type 2 myocardial infarction complicated with acute heart failure in a Sri Lankan female who presented on day 3 following a hump-nosed viper bite.

## Case report

This 39-year-old previously healthy female presented to the local hospital following progressive shortness of breath with associated orthopnea, paroxysmal nocturnal dyspnea, and bilateral lower limb oedema following a snake bite. A snake bite occurred 3 days ago. She was experiencing chest tightness on day 3 of the illness but denied any productive cough, fever, or muscle fatigue. She did not experience any neurological weakness or bleeding manifestations. She complained of reduced urine output since morning without the passage of dark-colored urine. She had a superficial wound in her right lower limb, which had mild cellulitis. The offending snake was identified as a hump-nosed viper after inspection by hospital-by-hospital staff.

On examination, she was afebrile, tachypneic, and tachycardic, with a respiratory rate of 20/min and a pulse rate of 120 bpm, respectively, and her on air saturation was 85%. She was hemodynamically stable, with a blood pressure of 100/60 mmHg. She had elevated jugular venous pressure with fine end inspiratory crepitations in the bilateral lower zones of the lung and bilateral lower limb oedema. Abdominal examination revealed a tender liver, which was probably secondary to congestion but no other organomegaly. Heart sounds were normal with no murmurs, and a neurological examination was also normal.

Her ECG showed ST segment depressions in the lateral leads (lead I, avL, and V4–V6), and the troponin I titer was 5.69 ng/mL. Whole blood clotting time at 20 min was normal. A chest X-ray showed evidence of pulmonary edema with cardiomegaly, Kerley B-lines, and hilar congestion. A full blood count revealed mild neutrophilic leukocytosis with a WBC of 15.61 × 10^3^, hemoglobin of 9.2 mg/dL, and platelets of 357. Her inflammatory markers were slightly elevated, with a CRP of 58 mg/dL. Her blood picture did not show any evidence of microangiopathic hemolysis, and her coagulation profile was normal, but it suggested the possibility of the thalassemia B trait. Her renal functions were normal, 0.8 mg/dL, and the urine full report was negative for cells and casts. Creatinine phosphokinase (CPK) was 169 U/L.

On admission, she was provided with supplemental oxygen to maintain saturation above 94% and was started on a frusemide infusion of 5 mg/h. She was catheterized and started on cardiac monitoring. Initially, with the history of reduced urine output, possible nephrogenic pulmonary oedema was suspected, and a nephrology opinion and urgent renal functions were traced. However, to our surprise, her renal functions were normal, and the ABG did not show significant metabolic acidosis. Ischemic changes in the ECG with a positive troponin titer suggested a possible cardiac pathology. Thus, she was started on enoxaparin since she did not have any bleeding diathesis and her coagulation profile was normal. In addition, she was started on oral antibiotics for the mild lower limb cellulitis.

The initial episode was managed as a non-ST segment elevation myocardial infarction with dual antiplatelets and anticoagulation. Heart failure was managed with loop diuretics. Since the antivenom available in Sri Lanka is not effective for hump-nosed viper bites, she was not given anti-venom. A 2D echocardiogram was performed, which revealed an ejection fraction of 60% with lateral wall hypokinesia, and her metabolic screening was normal. She underwent CT coronary angiography, since she was a low-risk patient, and she was found to have normal coronary arteries with no evidence of coronary thrombosis or embolism. This case was, therefore, concluded to be a case of venom-induced type 2 myocardial infarction complicated with acute heart failure secondary to a to a hump-nosed viper bite (Table 1).`

**Table 1:** Time line of events

Date	Events	Investigations	Interventions
2022/11/08	Hump nosed viper bite Hospital admission		Oral antibiotics and discharged
2022/11/11	Progressive shortness of breath with chest tightness (re-admission to local hospital) transferred to National hospital for further management	ECG—ST segment depressions and T inversions in lateral leads Troponin I titer + CXR—acute pulmonary oedema Coagulation profile/ renal functions—normal	S/C enoxaparin with dual antiplatelets and statin therapy Intravenous frusemide therapy and supplemental oxygen
2022/11/13	Reduction in oxygen demand	2DECHO—EF 60% with mild lateral wall hypokinesia	Converted to oral frusemide Enoxaparin omitted after 3 days of therapy
2022/11/15	Clinically well	CT—coronary angiogram performed normal study	
2022/11/17			Patient was discharged. Planned to review in 3 months at clinic

After the initial stabilization, she was continued on dual antiplatelets, beta blockers, angiotensin-converting enzyme inhibitors, and anti-anginal medications until the proper CT coronary angiography was available. Since she had normal coronary arteries in the CT coronary angiography, the patient was discontinued on antiplatelets and other medications she was on for the management of an acute coronary event. Her 2D echocardiogram was repeated in 3 months, which showed complete resolution of lateral wall hypokinesia with ejection fraction > 60%. She is waiting to undergo high-performance liquid chromatography (HPLC) for an assessment of her thalassemia status.

## Discussion

The hump-nosed viper, with its three main species found in Sri Lanka, including *Hypnale hypnale, Hypnale zara, and Hypnale nepa,* is widely distributed in Sri Lanka [1]*. Hypnale hypnale* is found almost everywhere in the country, except in Jaffna. *H. nepa* is found confined to the central hills, and *H. zara* is found in the south-western wet zone and in the central highlands. All three types of snake species are venomous. Even though all three species look the same superficially, with regard to scale count, they are different.

*Hypnale nepa* and *H. zara* species are endemic to Sri Lanka, while *H. hypnale* is also found in the Indian peninsular [1]. The majority of bites occur in the evening, and due to the short striking distance, most bites are in the extremities. In the literature, there is a diversity of events reported with regard to envenomation by hump-nosed viper bites, but the patterns of bites and features of systemic envenomation appear to be the same among different and larger series of studies, making it necessary to describe variations among the species [5].

The potency of the venom of all three species is similar, and it delivers the same degree of cytotoxicity, which is considered to be the most potent effect. Most effects of venom are due to phospholipase A2 activity. In addition, the venom of hump-nosed viper species has anticoagulant and procoagulant effects, leading to disturbances in homeostasis. Major organ-threatening manifestations can occur due to nephrotoxicity, myotoxicity, and neurotoxicity. This has been reported with the venom of the hump-nosed viper species. The hemolytic action of venom has also been described [6]. Administration of antivenom was not effective in treating severe local envenomation [6].

Clinical features of envenoming include nonspecific symptoms, such as abdominal pain, nausea, vomiting, fever, and headache. Local envenoming signs are seen in nearly 90% of patients, which include local pain, swelling, necrosis at the bite site, reginal lymphadenopathy, hemorrhagic blisters, etc. Common systemic manifestations include venom-induced consumptive coagulopathy and acute kidney injury. Rarely Snakebite-associated thrombotic micro-angiopathy, hemolytic uremic syndrome (HUS), thrombotic thrombocytopenic purpura (TTP), microangiopathic hemolysis, Kounis syndrome, purpura fulminans, generalized ecchymoses, pulmonary hemorrhage, intracerebral hemorrhage, acute ischemic infarction, coma, shock, severe diarrhea, and hyponatremia resulting in seizures are reported in the Sri Lankan context [7].

Cardiac manifestations are rather rarely reported in the literature with regard to hump-nosed viper bites. In a study that was done in 1999, neither of the cases revealed cardiac muscle involvement in association with hump-nosed viper bites [8]. In comparison to cases reported worldwide, Sri Lanka has a minimal number of case reports with regard to cardiac and cerebral toxicity, which may be due to differences in the composition of venom [8]. The exact mechanisms of cardiac manifestations are unknown. Thrombotic microangiopathic hemolysis [3], direct cardiac toxicity, venom-induced coronary artery spasm, hypovolemia due to anaphylactic shock or bleeding [9], toxin-induced myocarditis, electrolyte disturbances, and Kounis syndrome [10] are the possible causes for cardiac involvement in these case reports.

In our patient, the diagnosis of acute myocardial infarction is made according to the fourth universal definition of acute coronary syndrome based on the presence of symptoms, the dynamicity of electrocardiography, and a positive troponin I titer. The presence of echocardiographic findings compatible with the area of involvement in the ECG further supports the diagnosis of myocardial infarction. Her chest X-ray revealing evidence of acute pulmonary oedema with normal renal function supports the diagnosis of cardiogenic pulmonary oedema. The possibility of pre-existing atheromatous disease was excluded by the CT coronary angiography in this low-risk patient, which revealed normal coronary arteries. The absence of metabolic risk factors weakens the indication for an invasive coronary angiography; therefore, it was not performed. She did not have microangiopathic hemolysis in her blood, and her coagulation profile was normal, which excluded the possibility of venom-induced consumptive coagulopathy complicating thrombotic microangiopathy. Kounis syndrome associated with venom was excluded in our patient because she did not have any symptoms of a hypersensitivity reaction or eosinophilia. Therefore, the possible underlying pathologies for the occurrence of infarction could be contemplated as possible venom-induced coronary artery spasm or venom-toxin induced myocarditis. Delayed contrast-enhanced cardiac MRI is the gold standard imaging modality for differentiating myocarditis which was not done in our patient due to the unavailability of resources.

The occurrence of symptoms on day 3 of the illness further highlights our case, since most cases associated with viper bites usually present within hours of a snake bite [10]. Its worthy to note the possible cardiac toxicity associated with hump-nosed viper bites, even though they are rarely reported, and the possibility of late onset of complications, which makes the course of the illness unpredictable.

## Conclusion

This case report highlights the rare occurrence of myocardial infarction in the absence of thrombotic microangiopathy in a patient following a hump-nosed viper bite. The exact mechanism of myocardial injury and infarction is poorly understood, and further studies are required to understand the pathophysiology of the underlying mechanism to take the necessary steps in managing these patients successfully.

## Data Availability

The data are available from the corresponding author on reasonable request.
